# Two-Phase Globally Coupled Low-Density Parity Check Decoding Aided with Early Termination and Forced Convergence

**DOI:** 10.3390/s24216893

**Published:** 2024-10-27

**Authors:** Kun Zhu, Hongwen Yang

**Affiliations:** 1 School of Electronic Engineering and Intelligent Manufacturing, Anqing Normal University, Anqing 246003, China; 2 School of Information and Communication, Beijing University of Posts and Telecommunications, Beijing 100876, China; yanghong@bupt.edu.cn

**Keywords:** globally coupled LDPC code, local/global two-phase decoding, early termination, force convergence, low complexity

## Abstract

To enhance the decoding efficiency of Globally Coupled (GC) LDPC codes, we incorporated Early Termination (ET) and Forced Convergence (FC) into the local/global two-phase decoding algorithm to expedite the decoding process. The two-phase decoding scheme integrates the ET technique to halt unnecessary iterations in the local decoding phase while employing the FC technique to accelerate convergence in the global phase decoding. The application of ET technology in the local decoding of GC-LDPC codes will not cause performance loss as in traditional block codes and will cause considerable complexity gains. For a longer code length and larger convergence differences between nodes’ global codes, the FC technique operates more efficiently in global code than local code. Two variants are proposed for the ET scheme in the local decoding, namely ET-1 and ET-2. The initial variant, ET-1, predicts whether local decoding can be successful according to data characteristics and stop the local decoding iteration that is not expected to be successful in time. In the case of ET-2, the saved local iterations are transformed to global decoding equally. The results show that ET-1 saves considerable decoding time complexity and ET-2 improves the performance of the GC-LDPC code with the same decoding time complexity. The combined approach of ET-1 with FC reduces the decoding time complexity up to 42% at a low Signal Noise Rate region while maintaining its performance; ET-2-FC two-phase decoding saves approximately 25% decoding time complexity while improving the BER by about 0.18 dB and FER by about 0.23 dB.

## 1. Introduction

The Globally Coupled (GC) Low-Density Parity Check (LDPC) code has gained significant attention in recent years due to its distinctive structure and decoding algorithm [1,2,3,4]. This code consists of multiple individual local LDPC codes interconnected by a global coupling component, which enhances the size and connectivity of the code. The local codes can be decoded independently or participate in the decoding process of the entire GC-LDPC code. This unique feature gives rise to a specialized decoding algorithm known as local/global two-phase decoding [1]. Thanks to the two-phase decoding algorithm, parallel local decoding reduces the decoding delay. In terms of hardware implementation, the GC-LDPC code enables more efficient systems with a higher throughput (reduces the flash read latency by up to 43%) [2,5,6,7]. In the Multiple Input Multiple Output (MIMO) system, the GC-LDPC code improves throughput by four and five times for code rates of 0.49 and 0.66 [8]. To make the GC-LDPC code design more flexible, many papers provide several constructions [9,10,11,12]. To enhance the performance of GC-LDPC codes, ref. [13] proposes a protograph-based construction using Extrinsic Information Transfer (EXIT) charts, which outperforms previous GC-LDPC codes. Additionally, converting the general global coupling part to a tail-biting structure can further improve the code’s performance [14]. The implicit global coupling part is implemented using free-ride codes [15]. This type of GC-LDPC code achieves the same code rate as local codes while providing more than a 0.8 dB gain compared to local codes. In most cases, finite fields or finite geometry are employed for constructing Quasi-Cyclic (QC)-GC-LDPC codes with a limited girth of six. However, short cycles often result in trapping sets and lead to an error-floor phenomenon in LDPC codes with message-passing decoding [16]. By applying the Greatest Common Divisor (GCD) criterion, the girth of GC-LDPC codes can be extended from six to eight [17].

The Early Termination (ET) algorithm is a conventional approach used to reduce the time complexity of decoding processes [18,19,20,21,22]. The ET technology consists of two components: Early Give Up (EGU) and Early Success (ES) [21]. EGU aims to terminate the decoding process early when it fails to converge, thereby saving computational resources. ES, on the other hand, stops the decoding process once it has successfully converged to a valid result. The method for determining convergence plays a crucial role in achieving an optimal performance for the code. Studies by [20,21] focus on decision theory related to ET algorithms. Additionally, Receiver Operating Characteristic (ROC) curves are employed as useful tools for analyzing detector sensitivity and establishing accuracy criteria [21].

The Force Convergence (FC) technology, similar to ET, is commonly employed as a conventional approach for reducing the decoding delay [23,24,25,26,27,28]. By continuously monitoring the nodes in real time, FC proactively identifies and eliminates nodes that satisfy the convergence condition during subsequent iterations. In most scenarios, node reliability is assessed based on their respective amplitudes.

The ET technology was initially utilized in the local phase decoding in this paper. The proposed ET-1 decoding scheme achieves a reduction of 40–42% in decoding delay compared to the conventional two-phase decoding scheme in the low Signal Noise Rate (SNR) region, without any performance degradation. Additionally, the ET-2 decoding scheme enhances the code’s performance while maintaining the same level of decoding delay as the conventional two-phase approach. Subsequently, the FC scheme is employed for global phase decoding to further reduce the overall decoding delay. In the ET-1-FC decoding algorithm, the FC scheme demonstrates greater efficiency savings within waterfall regions while delivering a comparable final performance to other algorithms. Moreover, with respect to the Bit Error Rate (BER) and Frame Error Rate (FER) improvements of 0.18 dB and 0.23 dB, respectively, within waterfall regions, along with a 25% reduction in time complexity during such periods, these advancements are achieved by employing the ET-2-FC decoding algorithm. Consequently, it can be concluded that both proposed two-phase algorithms effectively enhance GC-LDPC codes’ overall efficiency under specific SNR conditions: whereas ET-1-FC focuses on avoiding the unnecessary iterations and reducing the decoding delay, ET-2-FC emphasizes improving efficiency and adaptability through the effective allocation of computational resources.

## 2. GC-LDPC Code

The GC-LDPC code consists of *t* local codes $H_{L}^{i},i\in \left[0,t-1\right]$, and the global coupling part is represented by $H_{X}$. The overall structure of the GC-LDPC code is illustrated in Equation (1):

(1) $$H_{GC}=\left[\begin{array}{c} \underline{\begin{array}{cccc} H_{L}^{0} & & & \\ & H_{L}^{1} & & \\ & & \ddots & \\ & & & H_{L}^{t-1} \end{array}}\\ \begin{array}{ccc} & & \begin{array}{ccc} & H_{X} & \end{array} \end{array} \end{array}\right].$$

This paper considers $H_{L}^{i}$ with the same size. Assuming *n* as the code length of $H_{GC}$, the local code length is n/t. The global coupling component results in a lower global code rate $r_{G}$ compared to the local code rate $r_{L}$.

### 2.1. Construct Method

Mostly, the GC-LDPC code $H_{GC}$ is constructed by deforming the well-designed (J,L) matrix [1,9,10,12]. For the Belief Propagation (BP) decoding algorithm, the structure of the LDPC code has a huge impact on performance, which includes the degree distribution and the characteristics of the cycle. The girth and degree distribution of $H_{GC}$ is determined by the (J,L) matrix. So, the design methodology for the (J,L) matrix is important within the construction process.

The previous construction methods can only guarantee GC-LDPC codes with a girth of six. In [17], we propose the GCD-based construction method to increase the girth to eight. The (J,L) GCD-based Full-Length Row Multiplier (FLRM) matrix is as follows:

(2) $$E=\left[\begin{array}{cccc} a_{0}b_{0} & a_{0}b_{1} & \cdots & a_{0}b_{L-1}\\ a_{1}b_{0} & a_{1}b_{1} & \cdots & a_{1}b_{L-1}\\ \vdots & \vdots & \ddots & \vdots \\ a_{J-1}b_{0} & a_{J-1}b_{1} & \cdots & a_{J-1}b_{L-1} \end{array}\right],$$

The integers $a_{i}$ and $b_{i}$, where $0\leq a_{0}<a_{1}<\cdots <a_{J-1}$ and $0\leq b_{0}<b_{1}<\cdots <b_{L-1}$, respectively, serve as exponential factors for the Cyclic Permutation Matrix (CPM) of size P×P. The row coefficient vector is denoted as $a=(a_{0},a_{1},\cdots ,a_{J-1})$ while the column coefficient vector is conventionally represented by $b=\left(b_{0},b_{1},\cdots ,b_{L-1}\right)=\left(0,1,\cdots ,L-1\right)$. In conventional GCD-based LDPC code, the minimum value of *P* is given by $P_{\min}^{*}=\left(a_{J-1}-a_{0}\right)\left(L-1\right)+1$. We discovered a new lower bound for GCD-FLRM to enhance the flexibility in designing GC-GCD-LDPC codes [29]. Deforming the well-designed GCD-FLRM as [29], the final GC-GCD-LDPC can be obtained. This paper focuses on analyzing GC-GCD-LDPC codes due to their superior performance compared to other types.

### 2.2. Local/Global Two-Phase Decoding

The local/global two-phase decoding scheme is the most interesting characteristic of GC-LDPC code. Each bit in the codeword belongs to both the local and global codes, enabling it to be decoded using both schemes. Suppose a codeword $v=(v_{0},v_{1},\cdots ,v_{t-1})$, where $v_{i},i\in \left[0,t\right)$ denotes the local codewords. Setting the responding received soft word as $r=(r_{0},r_{1},\cdots ,r_{t-1})$, the first step of local/global two-phase decoding is local phase decoding. Through *k* times of local iterations, while the local words $v_{0}^{k},v_{1}^{k},\cdots ,v_{t-1}^{k}$ satisfy each local check node constraint, we check the global coupling constraints. If $v^{k}$ satisfies all parity checks, end the decoding process and send $v_{0}^{k},v_{1}^{k},\cdots ,v_{t-1}^{k}$ as the output. For the iterations of local codes individually, parallel decoding saves *t* decoding delays compared to normal sequential decoding. Besides, during the local iterations, the global coupling part does not participate, and thus local decoding saves much computation complexity compared to the global phase decoding. When the channel environment is good, the local decoding saves much decoding delay compared to the conventional block decoding. The global phase decoding ensures the performance of the code in the harsh channel environment.

**Figure 1:**
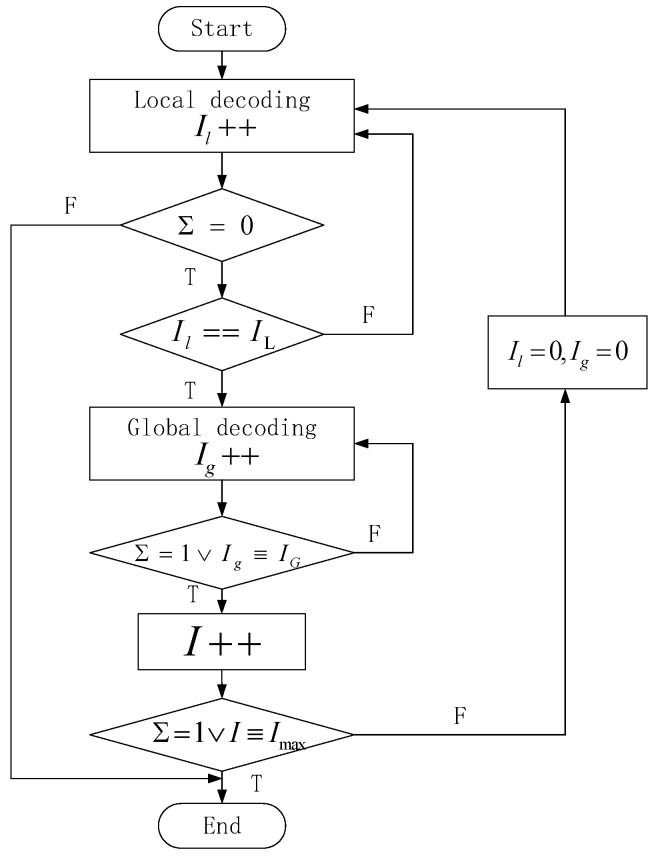
Local/global two−phase decoding.

The decoding flow chart is shown in Figure 1. In the figure, ·∨· denotes the logical relation of OR, T means TRUE, and F means FALSE. Let Σ=0,1 represent decoding nonsuccess or success, respectively. And $\hat{\Sigma }=0,1$ represents the predicted result of decoding nonsuccess or success, respectively. $I_{L}$ and $I_{G}$ define the maximum number of iterations for local and global decoding, respectively. Additionally, $I_{max}$ determines the maximum number of switches between the two phases. $\left(I_{L},I_{G},I_{max}\right)$ defines a two-phase decoding process. When $I_{max}=0$, the simplified version of this algorithm becomes a two-level decoding algorithm [2], which is determined by $\left(I_{L},I_{G}\right)$. For simplicity, this paper considers local/global two-level decoding as its chosen scheme.

As the introduction mentioned, local phase decoding saves much decoding computation complexity and delay in high SNR regions. However, in low SNR regions, local phase decoding is hard to decode individually. The unnecessary local iterations lead to meaningless decoding computation complexity. This paper gives two ET schemes to solve this problem. The computation complexity of global phase decoding is relatively higher, especially for the code with a large *t*. To further reduce the decoding delay of GC-LDPC code, we adopt the FC technology to the global phase decoding. A longer code length leads to greater differences in node information in global phase decoding, and FC technology can work more efficiently. The proposed ET and FC technologies focus on the decoding delay in low SNR regions. With the SNR rise, the probability of successful local decoding gradually increases, the judgment of ET does not work, and global decoding is barely needed.

## 3. Early Termination Algorithm

### 3.1. Decision Theory

In the initial ET scheme, the variable node reliability (VNR) is defined as Equation (3) [22]:

(3) $$VNR=\sum_{\forall i}\left|L_{i}\right|.$$

The $L_{i}$ is the log-likelihood ratio (LLR) of the $i^{th}$ variable node. Ref. [21] gives three indicators of judgment, which are defined as $v_{0}-v_{2}$ in Equation (4), to monitor the convergence behavior of iterative decoding:

(4) $$\begin{array}{cc} v_{0} & ={∥L∥}_{1}/n\\ v_{1} & =1-{∥s∥}_{0}/\left(n-k\right)\\ v_{2} & =|\{L_{i}:|L_{i}|>\theta \}|/n. \end{array}$$

where ∥·∥ denotes the norm and |·| denotes the absolute value. $v_{0}$ is the mean magnitude of the LLR of all variable nodes. $v_{1}$ is the fraction of satisfied check nodes, $s=\hat{x}\cdot H^{T}$, and $\hat{x}$ is the hard judge value of L. $v_{2}$ is the fraction of the large amplitude of the variable nodes, and the judgment threshold is θ.

Through the above three indicators, the respective criterion is designed as $C_{0}-C_{2}$.

*C* _0_
If $v_{0}^{l}<v_{0}^{l-1}$ appears *T* times, terminate decoding, $l<I_{L}$.
*C* _1_
If $v_{1}^{l}<v_{1,min},I_{l}>l_{1,min}$, the $v_{1,min}$ is the $v_{1}$ value in the $l_{1,min}$ iteration, terminate decoding. Or, when successive $v_{1}^{l}\leq v_{1}^{l-1}$ occur *T* times, stop decoding.
*C* _2_
If $v_{2}^{l}<v_{2,min}$ occurs when $I_{l}\geq l_{2,min}$, terminate decoding. $v_{2,min}$ is the $v_{2}$ value in the $l_{2,min}$ iteration.

$C_{0}$ and $C_{2}$ are both limitations of variable nodes, and $C_{2}$ works better than $C_{0}$. Thus, in this paper, $C_{1}$ and $C_{2}$ are utilized in local decoding in the meantime.

To choose the proper parameters in $C_{1}$ and $C_{2}$, Ref. [21] uses the ROC curve to select these parameters. The False Positive Rate (FPR) $P_{FP}$ and True Positive Rate (TPR) $P_{TP}$ are

(5) $$\begin{array}{c} P_{FP}=Pr\left\{\hat{\Sigma }=1|\Sigma =0\right\}\\ P_{TP}=Pr\left\{\hat{\Sigma }=1|\Sigma =1\right\} \end{array}$$

The ROC curve is the relation of $P_{FP}$ and $P_{TP}$ and is defined as

(6) $$P_{TP}:=f_{TP}\left(P_{FP}\right)$$

The area under the ROC curve $A_{ROC}=\int_{0}^{1}f_{TP}\left(p\right)dp$ is an effective quantitative judgment index. A larger $A_{ROC}$ indicates a more accurate prediction and a more reasonable setting of parameters. If the parameters are set perfectly, $A_{ROC}=1$.

### 3.2. ET Two-Phase Decoding

**Figure 2:**
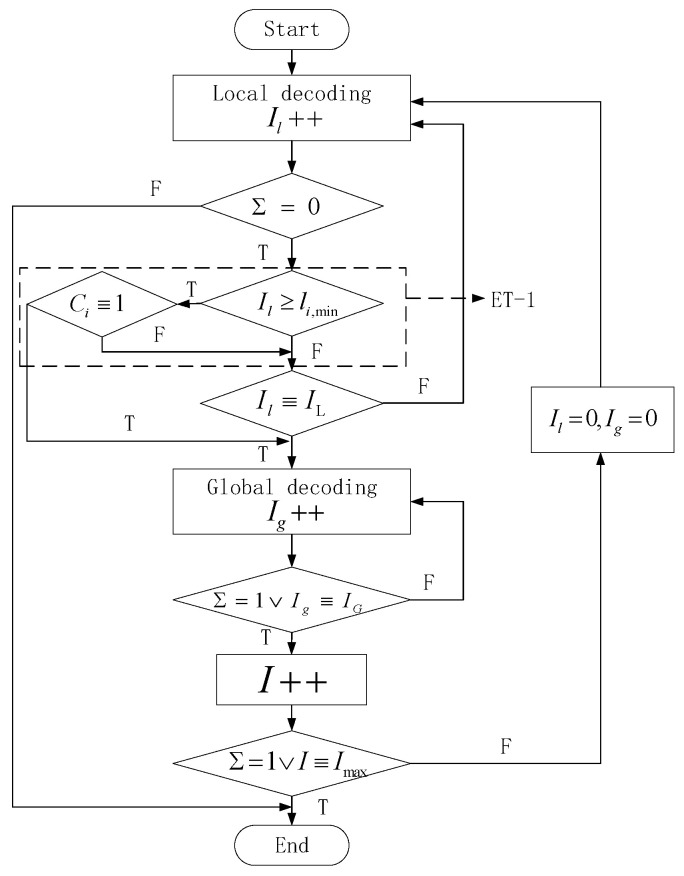
Flow chart of ET-1.

The purpose of introducing the ET scheme to GC-LDPC decoding in this paper is to reduce the unnecessary iterations of local decoding at a low SNR. Therefore, the ET scheme is only used for the local decoding process. Thus, we propose the ET-1 scheme as Figure 2.

The processes in the dotted box represent the ET decision. Well-designed parameters can significantly reduce unnecessary iterations of local decoding. In $C_{1}$, either $l_{1,min}$ or *T* serves as the key parameter. To facilitate the simulation, this paper adopts the latter approach. In $C_{2}$, selecting optimal values for both $l_{2,min}$ and $v_{2,min}$ is crucial for optimizing the ET scheme.

To allocate the decoding time complexity more reasonably, we propose ET-2 to assign the decoding time complexity adaptively. The number of decoding iterations saved by ET-1 translates into $I_{G}^{T}$, which is the additional number of global decoding iterations:

(7) $$I_{G}^{T}=\left\lceil \left(I_{L}-{\hat{I}}_{L}\right)\vartheta \right\rceil .$$

In this context, ${\hat{I}}_{L}$ denotes the actual iteration number at which local decoding is prematurely terminated. Due to the significantly higher complexity and delay associated with global decoding compared to local decoding, an additional parameter ϑ<1 is introduced in the above equation to regulate the overall decoding delay. The local codes in this paper are the regular degree $d_{L}$ and the global code is the regular degree $d_{G}$. The local codes are *t* times copies with the same size, and the local codes adopt parallel decoding. The rate of local/global decoding delay is $\vartheta =d_{L}/d_{G}/t$.

**Figure 3:**
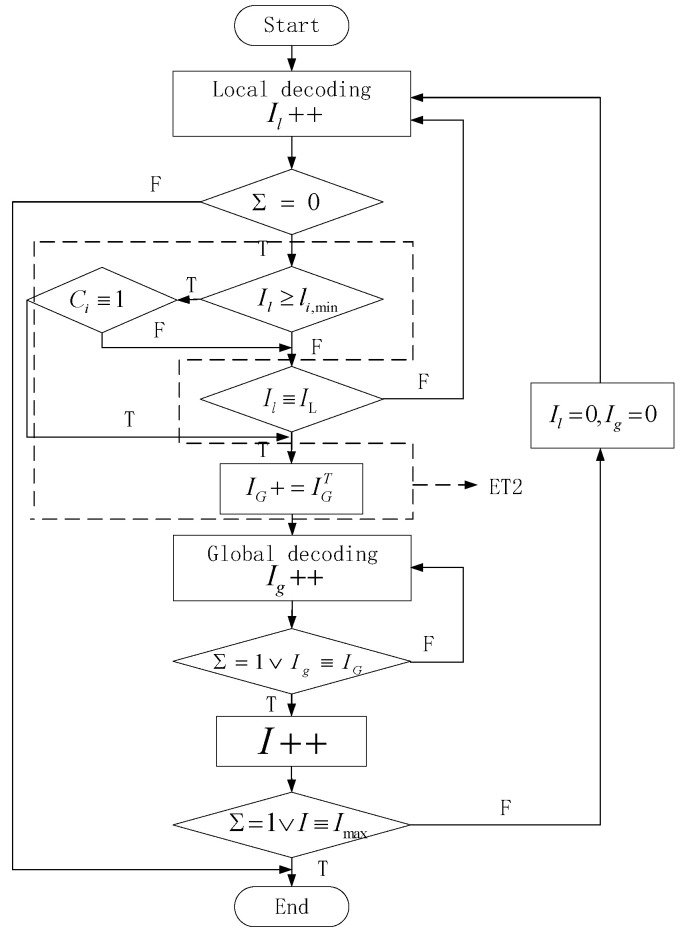
Flow chart of ET-2.

The process of ET-2 is shown in Figure 3.

In a low SNR region, the ET-2 two-phase decoding adaptively increases the number of iterations in global decoding. Thus, the performance in the low SNR region is effectively improved. It is worth mentioning that the ET scheme aims to improve the GC-LDPC code’s performance in a low SNR region. With the SNR increase, the impact of ET-1 and ET-2 decreases gradually.

The ET scheme avoids the unnecessary iterations of local decoding, especially in a low SNR region. The ET-1 directly stops the unnecessary local iterations to reduce the decoding delay. The ET-2 adaptively transforms the saved local iterations to extend the iteration of global decoding.

## 4. Force Convergence Algorithm

To further improve the decoding efficiency, the FC scheme is introduced into the two-phase decoding. In modern communication systems, the high-order Quadrature Amplitude Modulation (QAM) is a popular method to improve the throughput. For example, the 5G communication has expanded to 256QAM from 64QAM in 4G. The reliability difference between bits increases significantly with higher modulation orders. In the context of an actual communication system, this difference inevitably becomes more pronounced. During the iteration process of the LDPC code, bits with higher reliability tend to converge earlier than others. The termination of node iteration corresponding to the convergent bit in a timely manner can significantly alleviate the decoding time complexity. The well-designed FC scheme not only reduces the decoding time complexity but also marginally enhances the code’s performance.

The original conception of FC and the reliability of variable and check nodes are defined as Equation (8):

(8) $$R_{j,i}=\prod_{i^{\prime },i^{\prime }\neq i}sgn\left(Q_{i^{\prime },j}\right)\Phi \left(\sum_{i^{\prime },i^{\prime }\neq i}\Phi \left(Q_{i^{\prime },j}\right)\right)$$

$\Phi \left(x\right)=log\frac{e^{x}+1}{e^{x}-1}$, and $Q_{i,j}$ is the message exchanged through the $i^{th}$ check node to the $j^{th}$ variable node in the log domain. The aggregate message is $B_{i}$ for the variable node and $C_{j}$ for the check node:

(9) $$\begin{array}{cc} B_{v}^{i} & =L_{i}+\sum R_{j,i}\\ C_{c}^{j} & =\sum \Phi (|Q_{i^{\prime },j}|) \end{array}$$

The judgment threshold of the variable and check nodes are $t_{v}$ and $t_{c}$. When $B_{v}^{i}>t_{v}$ and $C_{c}^{j}<t_{c}$, the node is deactivated for the rest of the iteration. To simplify the judgment process, Ref. [17] uses the adjacent deactivated variable nodes to judge the check node instead of directly judging the check node information. This scheme is called Simplified Forced Convergence (SFC). In the MinSum decoding scheme, the check nodes’ operation in SFC is shown as Equation (10):

(10) $$C_{c}^{i,j}=\prod_{j^{\prime }\in \Psi_{i}\setminus j}sign\left(Q_{i,j^{\prime }}\right)\min_{j^{\prime }\in \Psi_{i}\setminus \left(j,k\right)}\left(\right|Q_{i,j^{\prime }}\left|\right)$$

$\Psi_{i}$ denotes the set of variable nodes adjacent to the $i^{th}$ check node, and *k* is the freezed variable node. The $i^{th}$ check nodes are only deactivated when the nodes in $\Psi_{i}$ are all freezed. Compared with the original FC, using the method of judging only variable nodes can significantly reduce the time complexity and memory accesses resulting in low power consumption [29]. Ref. [27] proposes an Adaptive Forced Convergence (AFC) algorithm. The dynamic threshold value is set as Equation (11):

(11) $$t_{v}=t_{0}-\left(\Delta t\times i\right)$$

Where Δt is the constant dynamic value of the threshold, *i* is the index of iteration, and $t_{0}$ is the initial threshold. To forbid the wrong judgment of nodes, the freezed check nodes, which unsatisfied the rest of the iterations, should be re-activated.

The size of the check matrix of the global code is much bigger than the local code, and global decoding has much more decoding delay than local code. The FC scheme utilizes the signal difference in global nodes to reduce the decoding time complexity. FC can be viewed as the ET at the bit level as it is very effective for global decoding, with large differences between nodes.

## 5. Simulation and Analysis

### 5.1. Code Construction

Firstly, we construct a GCD-based GC-LDPC code, which is called GC-1 [17]. Building a GCD-FLRM sized 4×13, the first three rows consist of the exponential matrix of local code, and the global part is built with three replications of the last row. The Z-factor of the exponential matrix is P=169. The code length is n=6591 and the code rate is r=0.751934.

### 5.2. ET Two-Phase Decoding

**Figure 4:**
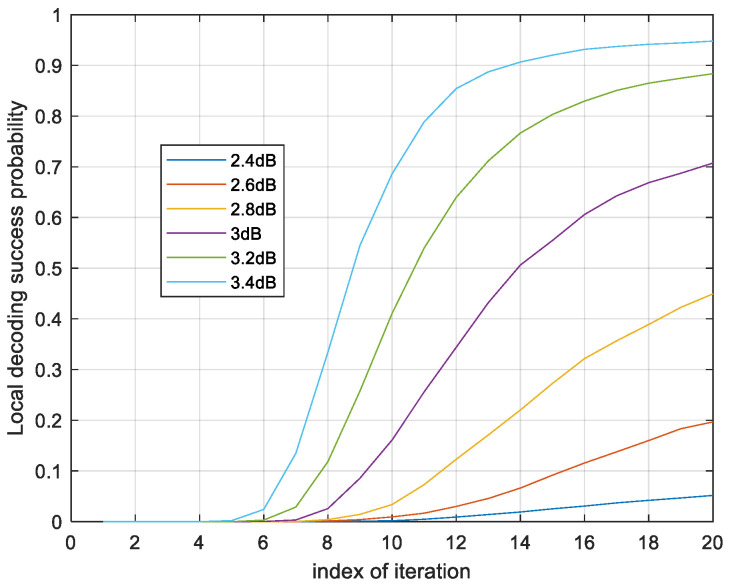
Probability of successful local decoding in 20 iterations with different SNRs.

Firstly, we use the ROC curve to optimize the parameters in the ET scheme. From Figure 4, the local code of GC-1 cannot decode successfully in four iterations. The $C_{2}$ is used in local decoding in four iterations. When the SNR is lower than 2.4 dB, the local code can hardly decode successfully by itself. The local phase decoding in this region is almost unnecessary. When the SNR is higher than 3 dB, the curve of success probability of the local decoding flattens out at 20 iterations.

**Figure 5:**
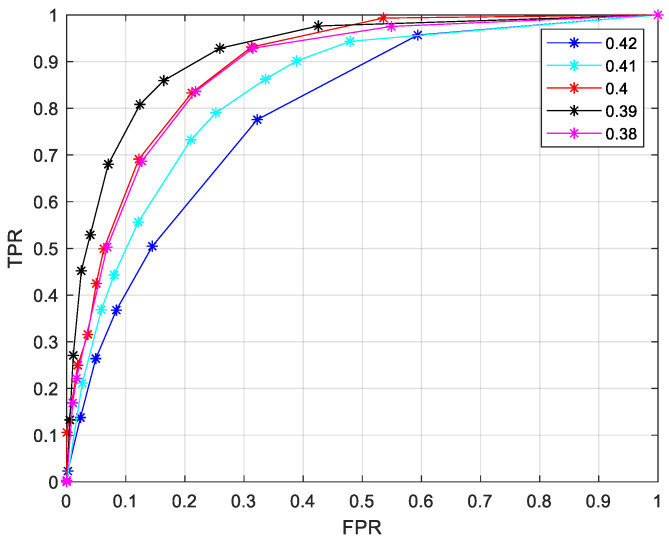
ROC curves in different values of $v_{2,min}$.

The local decoding has a probability of convergence after 10 iterations. Therefore, $C_{1}$ works with a large probability after 10 iterations. The ROC curves of $v_{2,min}$ are in Figure 5. The threshold of the hard decision is predefined as θ=5.

In Figure 5, it is easy to see $v_{2,min}=0.39$ is the best selection because it maximized $A_{ROC}$. $v_{1,min}$ and $v_{2,min}$ are optimized in the same way. To avoid repeating the elaboration of the optimization process, we give Table 1 as the summation of the ET schemes.

GC-1 is simulated in the AWGNC with QPSK modulation. For the sake of statistics, each time the node participates, the iterations account for one time complexity. In this paper, local codes are decoded in parallel, and the time complexity of local decoding is $L_{t}=L_{c}/t$. The maximum number of the local iterations is set as $I_{L}=40$. To make the global decoding have an equal decoding time complexity, the maximum number of global iterations is set as $I_{G}=10$.

**Figure 6:**
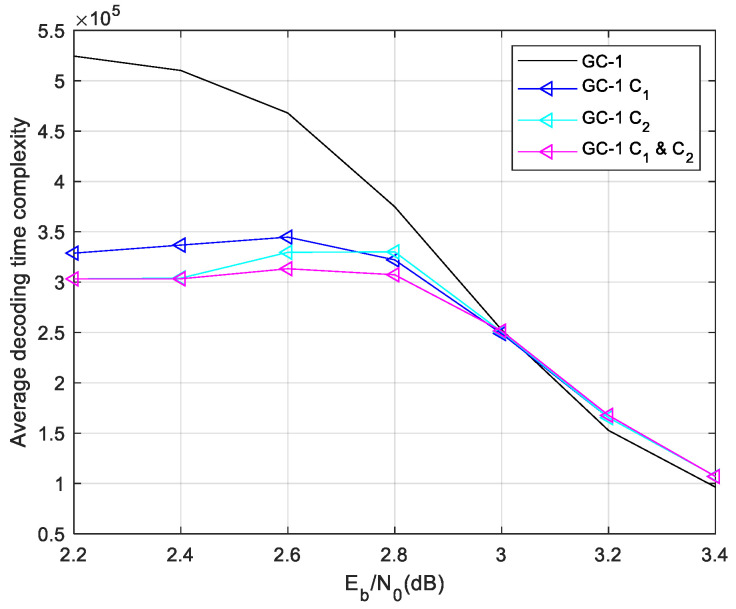
Complexity of GC-1 in different ET-1 schemes.

**Figure 7:**
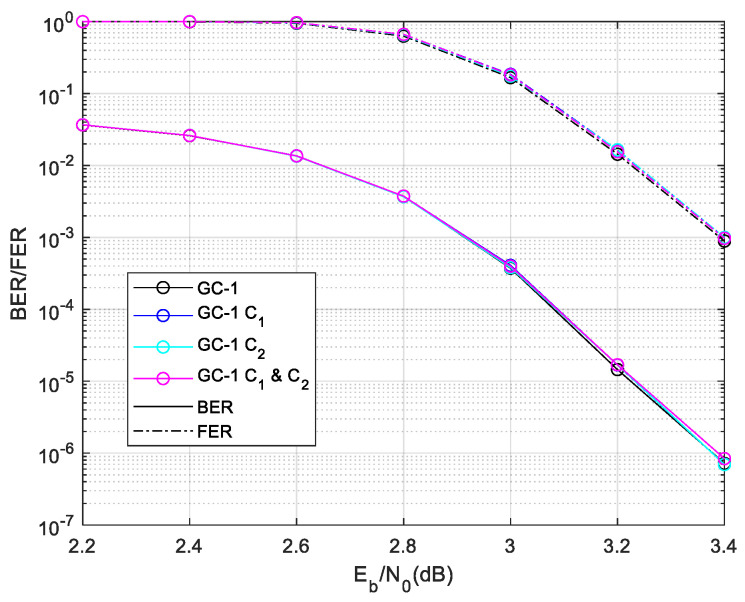
Performances of GC-1 in different ET-1 schemes.

Firstly, the ET-1 schemes are used for GC-1. The comparison result of the average time complexity of ET-1 is given in Figure 6. The results show that the ET-1 schemes save much complexity in a lower SNR region. In a higher SNR region, the ET-1 schemes have a similar time complexity with the normal local/global two-level decoding. This result demonstrates that the misjudgment probability of the designed ET scheme is extremely low. While the ET schemes’ misjudgment leads to the Early Termination of local decoding, thus entering global decoding, it will lead to a large increase in the decoding time complexity. The $C_{1}$ and $C_{2}$ scheme saves more decoding time complexity than others. While the SNR is 2.2 dB, the $C_{1}$ and $C_{2}$ scheme save 42.19% time complexity compared with regular two-level decoding. Then, we give the performance of GC-1 as Figure 7. The designed ET-1 schemes have a similar BER and FER performance with the normal local/global two-level decoding.

It is worth mentioning that the ET-1 scheme obtains a slightly higher time complexity in the high SNR region. This is because some codewords that can be correctly translated in local decoding are misjudged and enter the global phase decoding. With the SNR increase, the probability of the misjudgment decreases gradually. This phenomenon exists in the remaining ET-type decoding scheme.

The complexity statistic and performance demonstrate that the proposed $C_{1}$ and $C_{2}$ ET-1 scheme saves lot of decoding time complexity in a lower SNR region without performance degradation.

**Figure 8:**
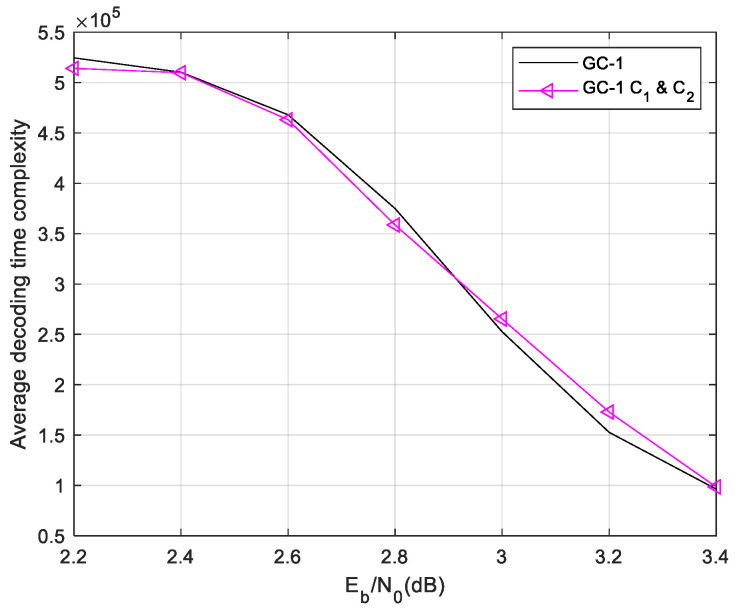
Decoding time complexity of GC-1 in different ET-2 scheme.

Afterward, we use the ET-2 in these codes. The proposed $C_{1}$ and $C_{2}$ scheme performs better than others, and the $C_{1}$ and $C_{2}$ scheme is no longer simulated in follow cases. The decoding time complexity of the ET-2 scheme is shown in Figure 8.

**Figure 9:**
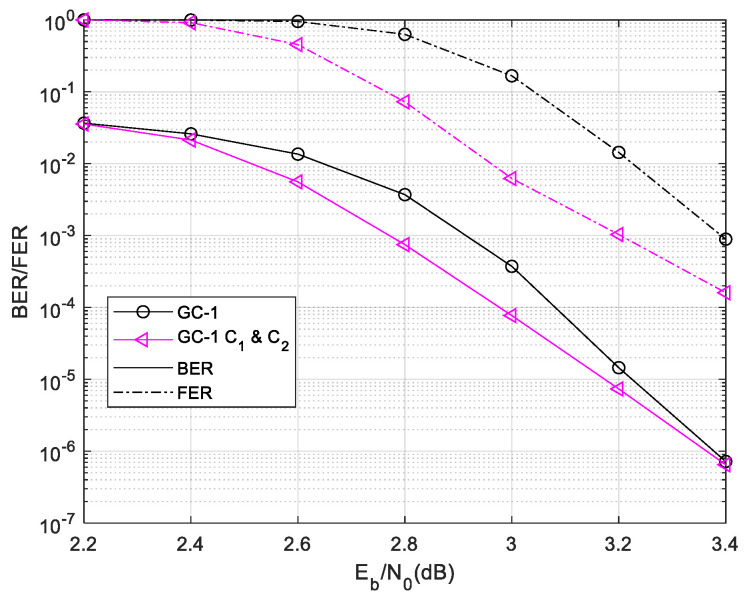
Performance of GC-1 in different ET-2 scheme.

The results indicate that the ET-2 scheme does not yield any reduction in decoding time complexity. The performance outcome is illustrated in Figure 9. The results demonstrate that the ET-2 scheme enhances GC-1’s BER by 0.18 dB and FER by 0.23 dB. In the low SNR region, there is a higher probability of failure in local decoding. The average value of $I_{G}^{T}$ is also higher. However, as the SNR increases, the average value of $I_{G}^{T}$ decreases. The performance of ET-2 based on GC-1 gradually approaches that of conventional decoding. This improvement in performance relies on limiting both the decoding time complexity and the number of global decodings performed. If the number of global decodings ($I_{G}$) is set to be sufficiently high, it becomes challenging for the ET-2 scheme to provide any further performance gain.

The ET-1 scheme significantly reduces the decoding time complexity without any degradation in performance. The ET-2 scheme enhances the decoding performance without introducing additional complexity, particularly when $I_{G}$ is constrained.

### 5.3. ET-FC Two-Phase Decoding

**Figure 10:**
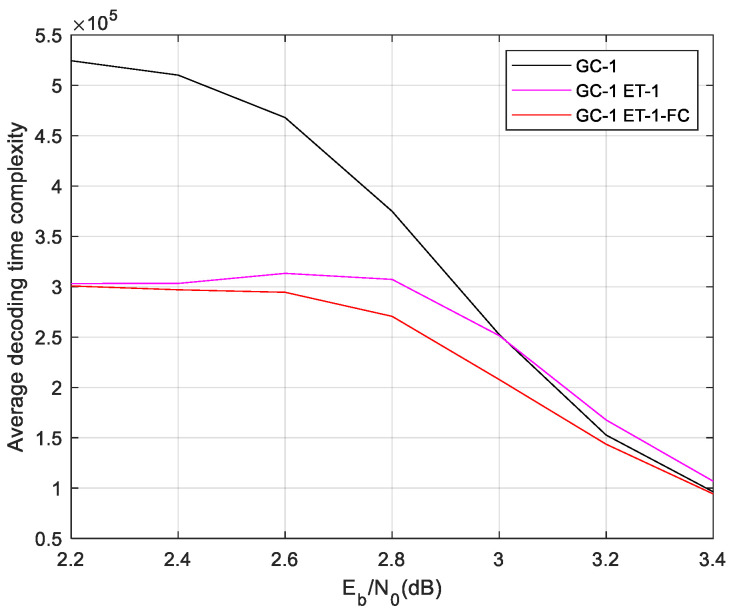
Decoding time complexity of GC-1 in ET-1-FC decoding scheme.

**Figure 11:**
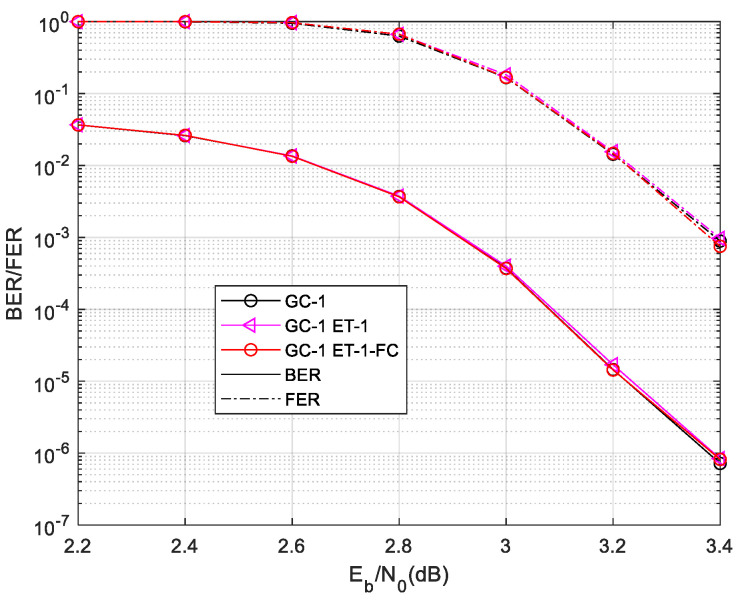
Performance of GC-1 in ET-1-FC decoding scheme.

To obtain a better decoding performance, we combine the ET and FC schemes in the two-phase decoding of the GC-LDPC code. In global phase decoding, the nodes converge better than the shorter local codes. So, the FC scheme is more suitable to be utilized in the global phase decoding process. In the proposed decoding algorithm, the ET-1 $C_{1}$ and $C_{2}$ scheme is utilized in the local phase decoding process and the AFC scheme is used in the global decoding process. The parameters of the FC scheme are optimized by the ROC curve as before. The final FC parameters are $t_{0}=20$δ=0.75. The performance and decoding time complexity are shown in Figure 10 and Figure 11.

The ET-1-FC decoding scheme offers even greater complexity reduction compared to the ET-1 scheme in most scenarios. In regions with lower signal-to-noise ratios, the convergence speed is slower and the impact of the FC scheme is less pronounced. However, in the waterfall region where the node convergence probability is higher, the FC scheme significantly reduces the decoding time complexity. Furthermore, the two-phase decoding algorithm of ET-1-FC does not introduce any performance degradation in terms of the BER/FER, similar to that of the ET-1 scheme.

**Figure 12:**
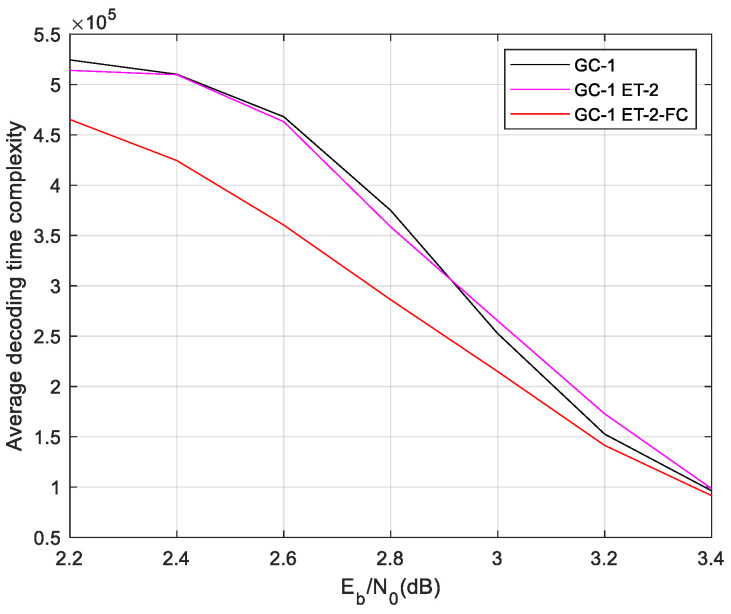
Decoding time complexity of GC-1 in ET-2-FC decoding scheme.

**Figure 13:**
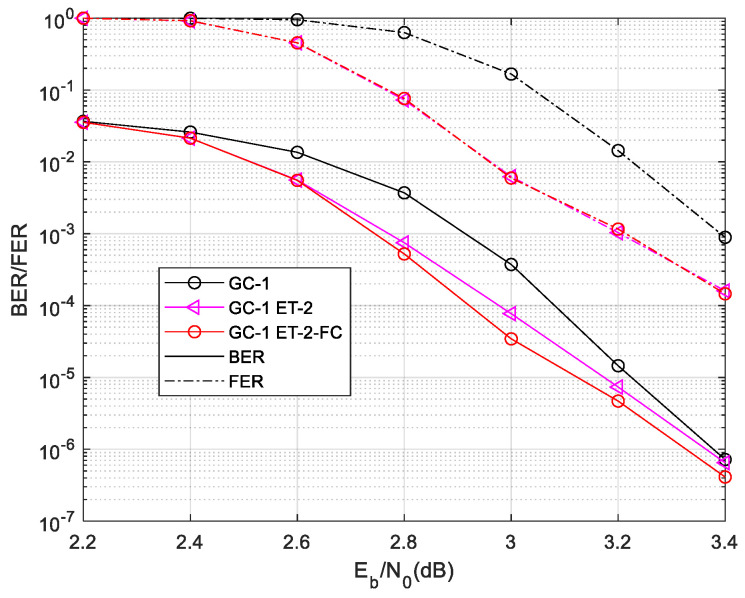
Performance of GC-1 in ET-2-FC decoding scheme.

The FC scheme is also employed in the ET-2 two-phase decoding algorithm. The complexity and performance of ET-2-FC two-phase decoding are illustrated in Figure 12 and Figure 13.

The ET-2 scheme extends the iterations of the global decoding, which leads the FC scheme to save more time complexity than the ET-1-based two-phase decoding. In the waterfall region, the ET-2-FC two-phase decoding algorithm saves about 25% decoding time complexity. And, the FC scheme also slightly improves the BER performance. The BER of ET-2-FC is slightly more improved than the ET-2 scheme. Because ET-2 extends the iteration of global decoding, FC technology has a deeper impact than bit-level convergence.

We simulated the GC-LDPC codes with a code length of 3000–12,000 and a code rate of 0.66–0.85. The results of these codes are similar to those with GC-1. To avoid repetition, the results of other codes will not be shown in this paper.

## 6. Conclusions

This paper introduces two novel two-phase decoding algorithms with the aim of further reducing the time complexity associated with GC-LDPC code decoding. The ET and FC techniques are incorporated into the decoding algorithm to minimize its computational time complexity. By judiciously applying these techniques, the proposed approach achieves a significant reduction in GC-LDPC code decoding time complexity without compromising performance. Specifically, the ET-1-FC decoding algorithm achieves a 42% reduction in time complexity within the low SNR region while maintaining performance integrity. On the other hand, the ET-2-FC algorithm enhances the BER/FER performance under constrained global iteration conditions, resulting in an improvement of 0.18 dB in the BER and 0.23 dB in the FER for this particular study case, accompanied by a 25% reduction in decoding time complexity within the waterfall region.

## Figures and Tables

**Table 1:** Optimized parameters of $C_{1}$ and $C_{2}$.

$C_{1}$	$C_{2}$
T = 1	θ=5
$l_{1}^{thr}=10$	$l_{2}^{thr}=4$
$v_{1}^{thr}=0.8$	$v_{2}^{thr}=0.39$

## Data Availability

The model of the subjects was public.
